# Mitochondrial DNA Variations in Colombian Creole Sheep Confirm an Iberian Origin and Shed Light on the Dynamics of Introduction Events of African Genotypes

**DOI:** 10.3390/ani10091594

**Published:** 2020-09-08

**Authors:** Herman Alberto Revelo, Diana López-Alvarez, Vincenzo Landi, Lauden Rizzo, Luz Angela Alvarez

**Affiliations:** 1 Department of Animal Science Animal, Faculty of Agricultural Sciences, Universidad Nacional de Colombia, Palmira 763533, Colombia; hareveloc@unal.edu.co (H.A.R.); laalvarezf@unal.edu.co (L.A.A.); 2 Department of Biological Science, Faculty of Agricultural Sciences, Universidad Nacional de Colombia, Palmira 763533, Colombia; 3 Department of Veterinary Medicine, University of Bari “Aldo Moro”, 70010 Valenzano, BA, Italy; vincenzo.landi@uniba.it; 4 Faculty of Livestock Science, Universidad Técnica Estatal de Quevedo, Quevedo 091050, Los Ríos, Ecuador; lgrizzoz@unal.edu.co

**Keywords:** Creole sheep, mitochondrial DNA, haplotype, maternal heritage

## Abstract

**Simple Summary:**

Creole sheep were introduced in South America by Spanish colonizers by the end of the fifteenth century. However, the exact dynamics of their formation remains uncertain. There is no consensus on the arrival of hair sheep of African origin, which may have reached the Caribbean coasts at different periods, compared to wool sheep that arrived from the Iberian Peninsula. Although bovines are the most important species for animal-origin food production in South America, sheep represent a strategic genetic resource for populations living in marginal areas with scarce economic resources. This study sheds light on the genetic structure of Colombian Creole sheep on the basis of mitochondrial DNA analysis. The main results indicate a common genetic layer originating from the first sheep that arrived during the European colonization, followed by the introduction of sheep of African origin.

**Abstract:**

The genetic origins and diversity of Creole sheep from five regions of Colombia were investigated based on mitochondrial DNA (mtDNA) variations across 89 sequences from five breeds: one wool Creole sheep (CL) and four hair Creole sheep, including Ethiopian (OPC_E_), Sudan (OPC_S_), Pelibuey (OPC_P_) and *Wayúu* (OPC_W_). A global comparison was done using 62 haplotypes from Iberian, African, Indian, Caribbean, Mexican, Caucasian and European sheep based on sequences retrieved from GenBank. This study aimed to identify the maternal origin of Colombian Creole sheep and their genetic relationships at a global level. The results showed 31 different haplotypes from Colombian Creole sheep, which can be assigned to maternal lineage B, the most common lineage found in European sheep breeds and the only one found in several Iberian breed (e.g., Churra, Spanish Merino) that most likely participated in the Creole formation. Additional analyses showed that wool and hair sheep retained a broad genetic identity despite being geographically separated. The global-level phylogenetic analysis revealed that Colombian Creole sheep belong to a distinct and defined genetic lineage that is likely the result of a founder effect with ecotypes of Iberian descent and the subsequent introduction of foreign breeds. This is consistent with historical reports on the presence of sheep in South America and, particularly, Colombia.

## 1. Introduction

Creole sheep in Latin America are derived from the introduction of Iberian sheep breeds during European colonization [1,2,3,4]. Normally, the term Creole (*Criollo* in Spanish) is used in animals and humans to refer to the descendant of an Iberian ancestor in the Americas. More recently, the term is used to identify livestock adapted to and originating from Latin America. According to empirical phenotypic comparisons, particularly based on wool characteristics [5], it is assumed that the introduced breeds were Manchega, Lacha and Churra [4]. It is also probable that other breeds such as the Spanish Castellana and Aragonesa, contributed to the creation of the Creole sheep in the Americas [6]. However, a clear breed classification for sheep did not exist in Europe at the time of the first introduction, with the exception of the Spanish Merino. However, this breed was unlikely to be used in the first livestock introduction due to a strict protection law of the Castilla kingdom (Honrado Concejo de la Mesta) [7]. Subsequently, hair sheep of West Africa were introduced during the slave trade and were intended to supply the labor demand of the sugarcane industry [8]. African breeds were widely crossed with those already established in America, due mainly to a better adaptation of African breeds to the tropical environment by specific traits acquired through natural selection, such as resistance to parasites, hardiness, prolificity, adaptation to hostile environments, food shortages and extreme temperatures [1].

In Colombia, Creole sheep play an important role in the family economy since they provide meat, wool and leather for local populations. Between 1960 and 1980, Creole breeds were widely crossbred with highly productive commercial breeds, mainly Suffolk, Hampshire Lacaune and Merino Precoce. Furthermore, in recent years, synthetic breeds, such as Dorper and Katadhin sheep, were also introduced, leading to a progressive replacement of the original population. As a result of these processes, the genetic component of Colombian Creole sheep is unclear, and reconstructing the history and genetic structure of its populations is complex. Wool sheep are mainly raised in the interior region of the country (i.e., Andean and southwestern regions of Colombia) on small subsistence farms. These sheep show a high capacity for animal fiber production and are a complementary component to agricultural activities and food security. On the other hand, hair sheep (or wool-less) may have originated from animals introduced during the colonization and, later, from imports arrived from the Horn of Africa, given the names that local people still use to define them (e.g., Sudan, Ethiopian and Abyssinian). Both wool and hair sheep are mainly raised in the Caribbean tropical region under traditional production systems in the absence of reproductive technology and control. Moreover, a group of hair sheep generally called Pelibuey (literally, “sheep with beef hair”) are raised mainly in southwestern Colombia together with sugarcane crops and are used for weed control. Additionally, one population of hair sheep is reared in the Guajira peninsula by the *Wayúu* indigenous population in a very arid and marginal territory. This paper provides the first description of this population.

Because there are several contact areas and numerous crosses have been made—most often without breeding criteria—the distinction between the different groups of sheep is unclear. In this scenario, there is a need to research the genetic basis underlying the current population of Colombian Creole sheep and compare it with the existing historical hypotheses. To address this issue, mitochondrial DNA (mtDNA) is very useful to track or refine highly complex patterns of migration and admixture, especially compared with autosomal markers [9,10,11], despite the fact that some studies have demonstrated a low power of discrimination of mtDNA between very phylogenetically close breeds and populations [12,13].

In this study, we report a genetic diversity and ancestry analysis of five Colombian Creole sheep breeds, followed by a comparison of the genetic relationships with other breeds from countries worldwide using the mitochondrial DNA control region sequence (D-loop). This research aimed to confirm the Iberian origin of Colombian Creole sheep and decipher the dynamics of the later introduction events from the African continent.

## 2. Materials and Methods

### 2.1. Ethics Statement

The Ethics committee of Universidad Nacional de Colombia authorized the project by which this research was conducted (process number P.OUN-006-19) and confirmed that no ethical approval was needed for this study. Blood samples were collected by qualified veterinarians during their routine practice within the framework of official programs aimed at the identification, health monitoring and parentage confirmation of the breeds and populations included in this study. The fieldwork did not involve any endangered or protected species and the study animals suffered no injuries. No other types of tissue (meat or other) were used in this study. Data derived from previously published studies were retrieved from public databases.

### 2.2. Sampling, DNA Preparation and Sequence Analysis

**Figure 1:**
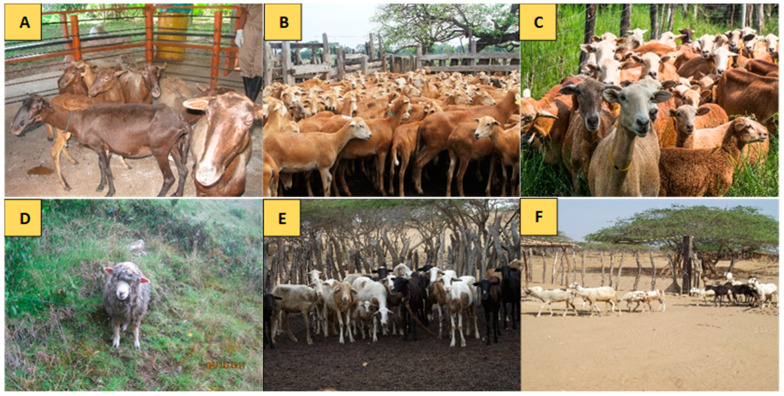
Five breeds of Colombian Creole sheep analyzed in the study. (**A**) Ethiopian (OPC_E_); (**B**) Sudan (OPC_S_); (**C**) Pelibuey (OPC_P_); (**D**) wool Creole sheep (CL); (**E**,**F**) *Wayúu* Creole sheep (OPC_W_).

**Figure 2:**
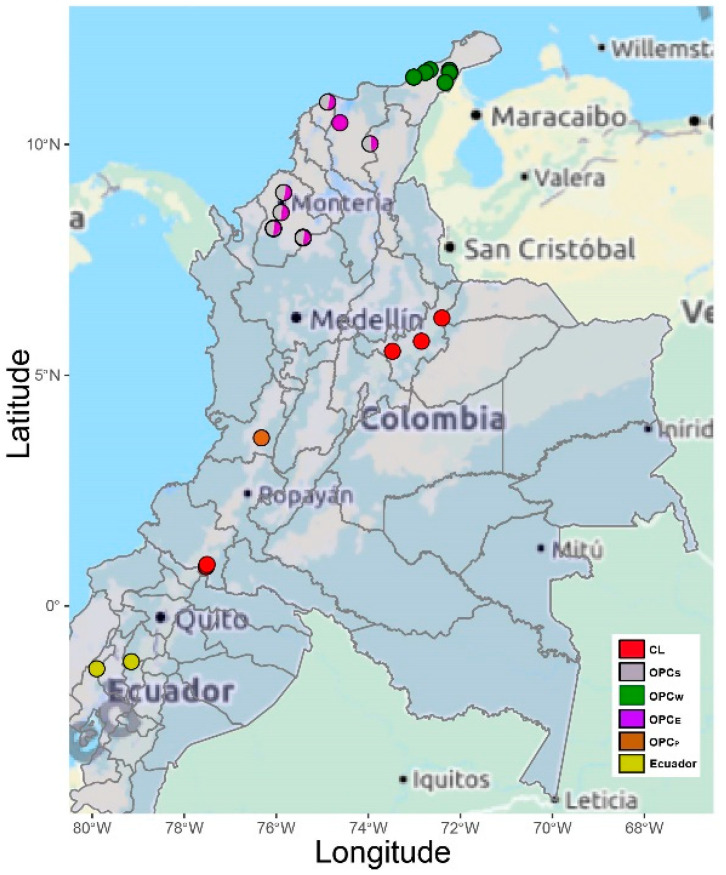
Map of the sampling locations of the five Colombian Creole sheep breeds and Ecuador sheep.

The scientific community in Colombia has proposed an acronym to describe Colombian Creole Hair Sheep as OPC (“Ovino de Pelo Colombiano” in Spanish) [14,15,16]. Five Creole sheep breeds were analyzed, including the wool Creole (CL) from Nariño and Boyacá regions; Ethiopian (OPC_E_) and Sudan (OPC_S_) Creole breeds in the regions of Córdoba, Cesar, Atlántico and Magdalena; Pelibuey Creole (OPC_P_) in Valle del Cauca; and *Wayúu* Creole sheep (OPC_W_) in La Guajira (Figure 1 and Figure 2, Table 1 and Table S1).

A total of 10 mL of blood from each animal was collected in BD vacuum tubes (Becton Dickinson, Franklin Lakes, NJ, USA) with k3-EDTA. Genomic DNA was extracted from 89 blood samples using the QIAamp^®^ DNA Mini Kit (QIAGEN, Hilden, Germany). DNA quantity and quality were evaluated by spectrophotometry using ColibriTM (Titertek Berthold, Neulingen, Germany) and agarose gel electrophoresis using a standard protocol to evaluate sample degradation. Additionally, three samples of Ecuadorian Creole sheep were included as representatives from the nearest country in the region.

### 2.3. PCR Amplification and Sequencing

The preliminary amplification of the ovine mitochondrial DNA control region comprised a sequence of 1428 bp between positions 15,346 bp and 157 bp of the complete circular mitochondrial sequence (NC_001941) using primers 15346for (5′-GGAGAACAACCAACCTCCCTA-3′) and 157rev (5′-TGATTCGAAGGGCGTTACTC-3′). The PCR reactions were performed on a Bio-Rad C1000 thermal cycler (Bio-Rad, Hercules, Hercules, CA, USA) using the following thermal profile: 95 °C for 5 min, 34 cycles of 95 °C for 45 s, 60 °C for 1 min and 72 °C for 2 min, with a final extension at 72 °C for 15 min. A total reaction volume of 25 µL was prepared using the Promega GoTaq kit (Promega, Madison, WI, USA), following manufacturer’s recommendations, and 0.1 µM of each oligonucleotide and 30 ng of DNA were added. The PCR products were visualized on a 1.2% agarose gel at 80 V for 30 min stained with GelRedTM (Biotium, Hayward, CA, USA). Next, the PCR products were purified using the ExoSAP-IT enzymatic system (USB Corporation, Cleveland, OH, USA) and sequenced by Macrogen (https://www.macrogen.com/) (Geumcheon-gu, Seoul, Korea). Sanger dideoxy-sequencing (in one direction) was triggered in position 15,393 bp by the internal primer 15393for (5′-ACTATCAACACCCAAAGCTG-3′). Additionally, 10 random samples were also sequenced in both directions using 15346for and 157rev primers to monitor the quality of the results. All oligos were synthesized by AlphaDNA (http://alphadna.com/) (Montreal, QC, Canada). 

### 2.4. Statistical Analysis

The electropherograms were visualized, edited and aligned using Geneious software v.8.05 (Biomatters Ltd., Auckland, New Zealand) [17]. Final sequence alignments were generated with the MUSCLE algorithm implemented in Mega 5.0 [18] using the reference sequence AF010406 [19], along with maternal reference haplotypes A (HM236174), B (HM236176), C (HM236178), D (HM236180) and E (M236182), described by Meadows et al. [20]. All new sequences were submitted to GenBank (accession numbers MT515537 to MT515620).

For global comparison, the 31 haplotypes found in Colombian Creole sheep were merged with representative haplotypes from several geographic regions (Europe, Africa, India and South America; Table S2). In the second alignment, noisy sequences and nucleotides with ambiguous sites were excluded, while the remaining sequences (Table S3) were aligned using default parameters in Mega 5.0 software [18]. Finally, 62 highly representative haplotypes from around the world were selected to construct the phylogenies together with the 31 Colombian haplotypes (Table S2).

Alignment conversion and input file preparation were achieved using Genalex v.6.5 (https://biology-assets.anu.edu.au/GenAlEx/Welcome.html) [21] and PGDSpider v 2.0.7.3 software (http://www.cmpg.unibe.ch/software/PGDSpider/) [22]. The estimation of nucleotide (π) and haplotypic (h) diversities, as well as the number and allocation of haplotypes, was determined using DnaSP v6 software (http://www.ub.edu/dnasp/index_v5.html) [23]. Furthermore, the expected heterozygosity (He), Wright’s fixation indices FST and an analysis of molecular variance (AMOVA) with 10,000 permutations were calculated using Arlequin v.3.5.2 (http://cmpg.unibe.ch/software/arlequin35/Arlequin35.html) [24].

The phylogenetic relationships among breed groups were inferred from the maximum likelihood optimization criterion using IQ-TREE v 1.6.8 (http://www.iqtree.org/) [25]. *Capra hircus* (AB004082) was established as an outgroup. The best nucleotide substitution model (HKY + G) was previously selected using jModelTest (https://github.com/ddarriba/jmodeltest2) [26] and support values for each node were calculated based on bootstrapping [27] with 200,000 replicates. Finally, the tree was visualized using FigTree v1.6.8 (https://github.com/rambaut/figtree/) and FaBOX web app (https://the-santos-lab.auburn.edu/srsantos/fabox/) was used to reduce the multiple alignments into single haplotypes with their respective frequencies.

Haplotype networks and structure phylogenetics were determined with Network 4.6.1.2 software (Network^©^ Copyright Fluxus Technology Ltd., Colchester, Essex, England) (www.fluxusengineering.com) using the neighbor-joining algorithm. Haplotype genealogies were identified using TCS (http://bioresearch.byu.edu/tcs/) [28] and tcsBU (https://cibio.up.pt/software/tcsBU/) [29] software, which allowed establishing the arrangement of haplotypic relationships between populations. First, the haplotype network was estimated in TCS v.1.21 (phylogenetic network estimation using statistical parsimony) and the output file was used as input to the online program tcsBU to estimate the haplotype genealogy or network.

## 3. Results

### 3.1. Genetic Diversity within Colombian Creole Sheep

A ~1K bp fragment between positions 15,489 and 16,294 of the mtDNA control region was selected for alignment to the reference sequence AF010406, defined by Hiendleder et al. [19]. In total, 53 polymorphic sites (S) were identified across 89 sequences of Creole sheep from Colombia including 17 singleton sites and 36 parsimony-informative sites (Table 2 and Table S3), important to classify haplotypes. We found 31 different haplotypes, as shown in Table S4.

**Figure 3:**
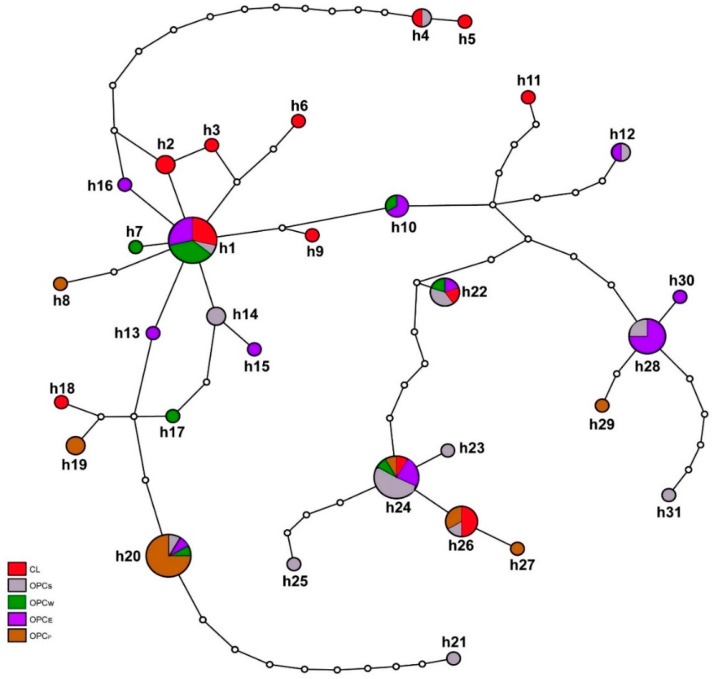
Pattern of 31 mtDNA control region haplotypes in Colombian Creole sheep constructed using the TCS program and plotted with tcsBU. Each haplotype is represented by a pie chart with size proportional to the number of sequences of each breed. The number of haplotypes corresponds to those indicated in Table 2 and Table S4. The small white circles indicate mutational changes. The five Creole breeds are indicated by the colors shown in the legend. CL—wool Creole; OPC_E_—Ethiopian; OPC_S_—Sudan; OPC_P_—Pelibuey; OPC_W_—*Wayúu*.

The nucleotide frequencies found in the haplotypes were 34.09% (A), 29.69% (T), 13.57% (C), 22.64% (G), and the G + C content was 0.362. The transition/transversion rate ratios were K1 = 26.21 (purines) and K2 = 57.26 (pyrimidines). We observed that the overall transition/ transversion bias is *R* = 20.29, determined by maximum composite likelihood [18,30]; this result shows that transitions are more frequent than transversions at the D-loop region. Table S4 and Figure 3 show the distribution of the 31 haplotypes found in the five Colombian sheep breeds (CL, OPC_E_, OPC_S_, OPC_P_ and OPC_W_). Nine haplotypes were shared among all breeds and 22 were unique (seven for CL and OPC_S_; four for OPC_E_; and two for OPC_P_ and OPC_W_).

The most frequent haplotype was h1, which was found in 14 individuals belonging to CL, OPC_E_, OPC_S_ and OPC_W_ breeds (Figure 3, Table S4), followed by the h24 haplotype that was shared among the five breeds (CL, OPC_E_, OPC_S_, OPC_P_ and OPC_W_) (Figure 2, Table S4). The h20 haplotype was the third most frequent with 12 individuals, mostly belonging to the OPC_P_ breed of Valle del Cauca (nine individuals) and in a lesser proportion to OPC_E_, OPC_S_ and OPC_W_. The fourth most frequent haplotype was h28, which was found in eight individuals of OPC_E_ and OPC_S_ breeds, including seven from the department of Córdoba and one individual from Magdalena. The fifth most frequent was h26 with six individuals shared among CL, OPC_S_ and OPC_P_ breeds, followed by h22 that was found in five individuals and was shared by CL, OPC_E_, OPC_S_ and OPC_W_ breeds. Among the less frequent haplotypes was h10 (*n* = 3) found in OPCS and OPCW (Figure 3, Table S4), while the remaining 22 haplotypes were specific variants of the different breeds (CL, 7; OPC_E_, 4; OPC_S_,6; OPC_P_,3; and OPC_W_, 2 (Figure 3, Table S4).

The expected heterozygosity (He) is considered the best estimator of genetic variability [31]. In this study, the highest values for He were found for CL and OPC_P_ (He: 0.28 ± 0.08), followed by OPC_E_ (He: 0.27 ± 0.1) and OPC_W_ (He: 0.26 ± 0.1), and the lowest value was displayed by OPC_S_ (0.22 ± 0.1). The genetic diversity indices showed that the lowest haplotypic diversity (h) was found in the OPC_P_ breed (0.59 ± 0.1), while the highest haplotypic diversity (h) values were found in CL (0.93 ± 0.04) and OPC_S_ (0.92 ± 0.04), followed by OPC_E_ (0.87 ± 0.04) and OPC_W_ (0.81 ± 0.1). The nucleotide diversity (π) showed the same trend as the haplotypic diversity (h) regarding the order of the highest and lowest values. In general, the haplotypic diversity (h) of the entire population was 0.92 ± 0.01 and the nucleotide diversity (π) presented values of 0.013 ± 0.001. Furthermore, CL and OPC_E_ breeds displayed the highest values of mean differences (K) between sequences (Table 2). The population structure was analyzed by an AMOVA, considering each breed as a group and showed 14.43% variation between groups (Fs_T_ = 0.14; *p* < 0.001). The Fs_T_ index was low yet significant, enough to differentiate between breeds, while the highest genetic variance was expressed within populations, indicated by a percentage of 85.57% (Table S5). We found differences between OPC_P_ and the other breeds (Fs_T_ ~0.20–0.32; *p* < 0.05), among OPC_W_ and OPC_S_ (Fs_T_ = 0.15; *p* < 0.05), OPC_W_ and OPC_P_ (Fs_T_ = 0.20; *p* < 0.05) and finally among OPC_E_ and CL (Fs_T_ = 0.07; *p* < 0.05) (Table S5).

### 3.2. Genetic Structure of Colombian Creole Sheep

**Figure 4:**
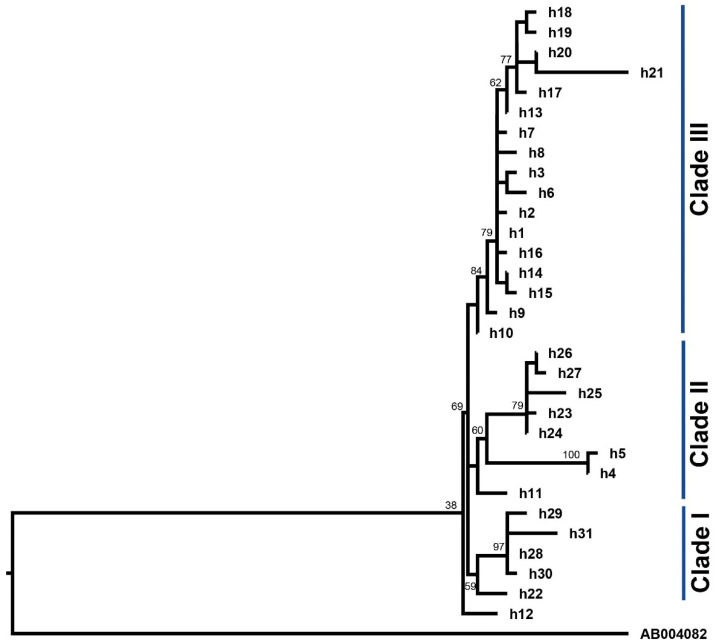
Phylogenetic tree inferred from maximum likelihood based on the analysis of 31 haplotypes found in five breeds of Colombian Creole sheep. *Capra hircus* (AB004082) was used as outgroup.

The phylogenetic reconstruction inferred from maximum likelihood (Figure 4) allowed separating the 31 haplotypes into three clades. We observe that haplotype h12 may be the most ancestral shared by two individuals of two breeds (OPC_E_, OPC_S_) (Figure 3 and Figure 4). The first clade comprised 16.13% of haplotypes, including three that were specific to Creole hair sheep breeds OPC_P_, OPC_S_ and OPC_E_ (e.g., h29, h31 and h30, respectively). Additionally, within this clade, haplotypes h22 and h28 were shared by at least two breeds (Figure 4, Table S4). The second clade was represented by 25.8% of haplotypes, including four that were breed-specific (e.g., h23, h25, h27 for OPC_S_ and h5, h11 for CL), one haplotype (h24) that was shared among the five breeds (OPC_E_, OPC_S_, OPC_W_, OPC_P_ and CL), and two haplotypes (h4 and h26) that were shared by at least two breeds. The third clade comprised 54.83% of haplotypes, including h1 that is shared by four breeds (CL, OPC_E_, OPC_S_ and OPC_W_) and the h20 haplotype that is mostly found in individuals of OPC_P._ Furthermore, within this clade, 13 haplotypes were breed-specific, for example, h14, h21 (OPC_S_), h7, h17 (OPC_W_), h13, h16, h15 (OPC_E_), h19, h8 (OPC_P_) and h18, h6, h9, h3 (CL) (Figure 4, Table S3). None of the clades showed any relationship between haplotypes and geographical distribution (Figure 4, Table S4). The branching pattern of the haplotypes showed that most are shared among the five breeds, although unique haplotypes also exist. In general, all branches were supported by high bootstrap values (BS > 50). The genetic relationships of the 31 haplotypes found in Colombian Creole sheep were compared with the five maternal lineages (A, B, C, D and E) reported by Meadows et al. [20]. The results showed that the haplotypes are related to the reference sequence HM236176 of haplogroup B (Figure S1). Furthermore, no relationships were found with the reference sequences of haplotypes A, C, D and E, described by Meadows et al. [20] (Figure S1).

### 3.3. Phylogenetic Analysis

To visualize the genetic relationships and the possible maternal origin of Colombian Creole sheep, the 31 haplotypes from Colombia were compared with 62 representative haplotypes that were retrieved from GenBank [3,4,12,13,19,32,33,34,35] and are described in Table S2. In addition, the reference haplogroup sequences defined by Meadows et al. [20] (HM236174.1, HM236183.1) were included in the analysis to correctly identify the top five haplogroups (A, B, C, D, E) of mtDNA.

**Figure 5:**
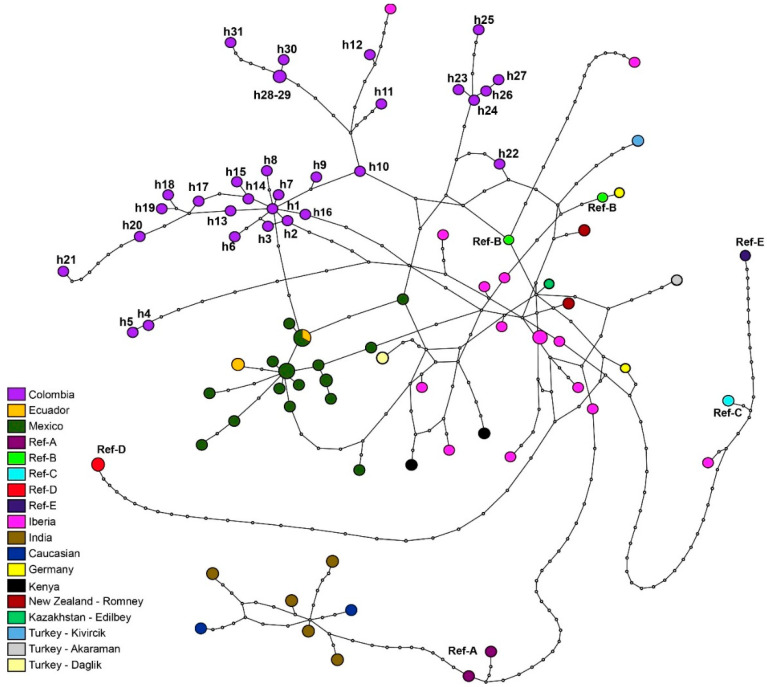
Pattern of 31 mtDNA haplotypes in Colombian Creole sheep and their genetic relationships with 62 reference haplotypes (Table S2). Each haplotype is represented by a circle with size proportional to the number of shared sequences; the small white circles indicate mutational changes and each country or breed are color-coded according to the legend. Reference lineages are listed as Ref, while the Colombian haplotypes are listed h1–h31.

The topology of the haplotype network allowed the identification of the relationship of the 31 haplotypes from Colombia with sequences of sheep from around the world (Figure 5). A close relationship of several haplotypes from Colombia with Iberian sheep was established; for example, haplotype h12 is composed of two sequences from OPC_E_ and OPC_S_ and differs by three mutational events from an Iberian haplotype (DQ491622) that belongs to haplogroup B described by Pereira et al. [13].

On the other hand, the h1 haplotype differs by two mutational events from the haplotypes in Mexico (MX11), described by Alonso et al. [4] and the E17 haplotype from Ecuador, which was generated in this study. Moreover, the h22 haplotype differs by seven mutational events from domestic sheep from Europe (Kivircik; AY091495) and by five mutational events from the reference sequence (AF010406) and the Merino Landschaf breed (AF039577) described by Hiendleder et al. [19]. Similarly, this same haplotype (h22) differs by five mutational events from the commercial Romney breed (Z35228) described by Wood & Phua [36]. The East African sequences (KT867771), described by Resende et al. [34] and the reference sequence of haplogroup B (HM236176) are also contained within this group, displaying two mutational events. Nine haplotypes (e.g., h1, h2, h3, h6, h7, h13, h14, h15, h16) of Colombian Creole sheep were found close to the haplotypes from Mexico described by Alonso et al. [4], differing by only two mutational events (Figure 5).

## 4. Discussion

Historical reports [1,37] and molecular marker-based studies suggest that Creole sheep in the American continent originated from sheep from the Iberian Peninsula that arrived during the European colonization between the 15th and 16th century [2,4,14] and sheep introduced from West Africa. The time and origin of the arrival of African sheep is not entirely clear. They probably originated from imports carried out in the 18th century along the slave route, but it cannot be excluded that some hairless sheep were acquired in the first ocean voyages from the Canary Islands. However, Primo [38] reports in his writings that from the analysis of historical texts it appears that the Creole animals originated from a few animals imported in the early years [8,38,39]. The native wool sheep that currently inhabit South America probably have maternal origins in Churra sheep [40] and the Merino breed imported from the Iberian Peninsula [41]. Delgado et al. [1] suggest that a few animals were brought from the Iberian Peninsula, which gave rise to multiple breeds in the Caribbean and, later, in the rest of the continent. Aguilar-Martínez et al. [42] propose that the existing hair sheep breeds in America are probably a mixture of animals from the Canary Islands and the subsequent introduction of sheep from West Africa. Particularly, the Canary Islands, inhabited by indigenous populations from Africa [15,43] and conquered by Spaniards between 1402 and 1496, constituted the first stop in the Atlantic ocean sailing [37]. Overall, the processes of genetic drift contributed to consolidating the breeds that currently exist on the South American continent.

However, the complete events of the arrival of the first hair sheep remain unclear. It is also assumed that some animals arrived through the trans-Atlantic slave trade routes from the African continent [44] used by Magdalene merchants who marketed with Aruba and Curacao and smugglers who traveled between the Caribbean islands and La Guajira [16]. Other introductions of hair sheep to Colombia date back to 1940 when a group of red African sheep (Ethiopian) from Abyssinia (today Ethiopia) were introduced to Armero, Honda and Venadillo, where their descendants remain [16]. In Colombia, there are few studies on the genetic diversity of Creole sheep, the majority focused on analyzing the nuclear genome (e.g., Vivas [44], Jiménez et al. [45], Ocampo et al. [14]) through microsatellite markers. This research is the first to analyze mitochondrial DNA variations to assess the genetic diversity of Colombian Creole sheep and obtain information about their maternal origin; furthermore, we also present the first molecular results described for the indigenous breed *Wayúu*, which constitutes part of the cultural identity of its people and an invaluable resource for their food security, since they live in the desert of La Guajira at extreme temperatures

The phylogenetic analysis based on 89 sequences of the D-loop region of Colombian Creole sheep revealed 31 haplotypes that are widely distributed throughout the country. In general, the haplotypic diversity (h) was high (e.g., 0.93, 0.92), while OPC_P_ presented the lowest value (h) 0.59 (Table 2). Most of the haplotypes were shared between wool and hair Creole sheep; particularly, the h1 haplotype was the most frequent and it was shared among four breeds, except for OPC_P_ (Figure 3, Table S4). This finding may allow the inference that Colombian Creole sheep breeds are in the process of expanding with respect to a more frequent lineage. On the other hand, 22 haplotypes were breed-specific (Figure 3, Table S4) and may have possibly originated from mutations that occurred after the European introduction or crosses of Creole sheep with commercial breeds; conversely, these variations may correspond to ancestral haplotypes from different lineages and contributed to the formation of Colombian Creole sheep. Currently, wool Creole from Boyacá (BCL) and Nariño (NCL) are found mainly in the high and cold areas of the country and are geographically separated from hair Creole sheep (OPC_E_, OPC_S_, OPC_P_ and OPC_W_) located in the low and warm areas; however, these sheep share common haplotypes (Figure 3, Table S4). Although the phylogenetic analysis (Figure 4) allowed separation of the 31 haplotypes into three different groups, the branching pattern of the haplotypes does not show consistency with geographic distribution; these results suggest a genetic closeness between wool and hair sheep regardless of the geographic location and this is consistent with a microsatellite marker analysis reported by Vivas [44], which showed common groups between wool (CL) and hair (OPC_E_, OPC_S_) Creoles reared in different regions.

The reconstruction of the phylogeographic history of five Colombian sheep breeds (Figure 5; Figure S2) indicated that all haplotypes converge into a unique and distinct phylogenetic node. This is likely due to a founder effect, in which an ancestral population constituted the genetic basis of the Creole sheep population in Colombia today. In addition, the presence of maternal lineage B demonstrates a common genetic component with Iberian ancestors at the mitochondrial DNA level. Specifically, this is supported by the presence of the h12 haplotype, which differs by three mutational events from the Iberian haplotypes (DQ491622) described by Pereira et al. [13] (Figure 5). According to Ferrando et al. [46], maternal lineage B, also called European, is common in the so-called Churro, Merino and Entrefino trunks (i.e., a classification according to the wool types frequently used in Spain), which possibly represent the genetic basis of many current Creole sheep in America [4]. Similarly, Pedrosa et al. [12] also reported the presence of lineage B, although they also found haplogroups A and C at low frequencies. On the other hand, nine haplotypes from Colombia (e.g., h1, h2, h3, h6, h7, h13, h14, h15, h16.) displayed close genetic relationships with most haplotypes found in Creole sheep from Mexico, described by Alonso et al. [4] and the E17, E18, E7 haplotypes from Ecuador generated in this study (Figure 5). Moreover, Campos et al. [47] also reported maternal lineage B in Mexican Creole sheep, that being the most frequent haplogroup in Spain and in Europe, which could demonstrate commonalities in the origin that connects the Creole sheep of Latin America with the Iberian sheep. The small number of animals introduced during the first years of the colony [38] suggests that genetic drift has an important role in the conformation of haplogroup frequency. The h22 haplotype differs by seven mutational events of Kivircik from Turkey (AY091495) and five mutational events from the Merino Landschaf breed haplotype (AF039577), described by Hiendleder et al. [19] and Romney sheep (Z35228) described by Wood & Phua [36]. The haplotypes of Kenya from East Africa (KT867771, KT867681), described by Resende et al. [34], were the farthest from the Colombian haplotypes (differing by 10 mutational events), although the former belong to haplogroup B. The differences due to the number of mutational events between African and other European breeds from the Colombian ones is higher when compared with other Creole sheep (Ecuador, Mexico). The same result is repeated with the Iberian breeds.

The haplotype network (Figure 5) reconstructed in the analysis of 31 haplotypes of Colombia and 62 haplotypes of several regions of the world showed a star-shaped pattern in which maternal lineages (A, B, C, D and E) described by Meadows et al. [20] are clearly differentiated. These results were supported by the phylogenetic analysis based on maximum likelihood (Figure S2). Colombian Creole sheep do not show haplotypes of maternal lineages A and C, contrary to what was expected from our initial hypothesis based on the presence of these lineages in sheep from the Iberian Peninsula [12,13] and West Africa [3] at low frequencies. These lineages are spread across African breeds. Indeed, both haplogroups A and B are found in Ethiopian sheep [48]. Likewise, in Sudan sheep, Gornas et al. [49] found that most sequences belonged to haplogroup B and only about 10% corresponded to haplogroup A. Moreover, in Argentina, Peña et al. [2] reported the presence of haplogroup A in four Creole breeds representative of four regions (e.g., Buenos Aires, Corrientes, Santiago del Estero and Salta). In this context, the absence of haplogroup A in Colombian Creole sheep may suggest that following the introduction of the first wool sheep, the introduction of hair sheep was done using males by substitution crossbreeding. The absence of haplogroups A and C, despite their presence in the Iberian Peninsula, could also be the result of dynamics of genetic drift due to the reduced number of animals introduced in the early years of colonization.

Finally, it is assumed that OPC_E_ and OPC_S_ breeds have an African origin (Ethiopia) so the presence of maternal lineage A was expected at least in low frequency, as reported in Adhena [48] and Gornas et al. [49]. The results found here indicate that there is no close genetic link between Colombian hair Creoles and possible ancestors from East Africa (Ethiopia). In this context, the genetic basis of Creole sheep probably originated from the first imports of livestock during the Spanish colonization, whereas the subsequent introduction of hair sheep from West Africa was perhaps done exclusively with male individuals, as reported by Miretti et al. [10] in cattle. In this regard, the analysis of autosomal variation in future studies could probably resolve this point.

## 5. Conclusions

This study provides the first phylogenetic analysis based on mitochondrial DNA D-loop variations in Colombian Creole sheep. Creole wool and hair sheep breeds were found to share a maternal genetic background and displayed genetic relationships with Iberian and African ancestors. These findings are consistent with historical reports on the presence of sheep in South America and suggest that Colombian Creole sheep are probable descendants of Iberian trunks with ancestry in ecotypes of West Africa and a subsequent introduction of commercial lines, although it is not clear if a particular breed was involved.

Since the same history of colonization is shared throughout South America, this particular maternal origin for Colombian Creole sheep could also be present in other populations of Creole sheep in Latin America. For instance, this study demonstrated that Creole sheep from Ecuador share the same common ancestor with Iberian sheep. Overall, this work contributes to characterizing Creole sheep in Colombia by defining their maternal origins, establishing global phylogenetic relationships and revealing the importance of these sheep as an animal genetic resource that must be conserved given their role in the rural economy.

## Figures and Tables

**Table 1:** List of Colombian Creole sheep breeds, typologies and geographical positions.

Breed	Acronym	Type	*N* (*f*)	Department (region)
Ethiopian	OPC_E_	Hair sheep	23 (6)	Córdoba, Cesar, Atlántico, Magdalena (Caribbean coast)
Sudan	OPC_S_	Hair sheep	23 (5)	Córdoba, Cesar, Atlántico (Caribbean coast)
Pelibuey	OPC_P_	Hair sheep	14 (1)	Valle del Cauca (Pacific Coast, Colombian southwest)
Wool Creole	CL	Wool	18 (8)	Boyacá; Andean region and Nariño (Colombian southwest)
*Wayúu*	OPC_W_	Hair sheep	11 (7)	Guajira Peninsula (Caribbean coast)

*N*—number of sampled animals; *f*—number of herds considered.

**Table 2:** Haplotype distribution and frequency in Colombian Creole sheep. n—number of animals; S—number of polymorphic sites; NS—Singleton’s number; Pi—parsimony-informative sites; K—mean number of nucleotide differences between sequences; h—haplotypic diversity and its standard deviation; π—nucleotide diversity and its standard deviation. CL—wool Creole; OPC_E_—Ethiopian; OPC_S_—Sudan; OPC_P_—Pelibuey; OPC_W_—*Wayúu*.

Breed	n	S	NS	Pi	K	h-SD(h)	π-SD(π)
**CL**	18	28	9	19	7.85	0.93 ± 0.04	0.011 ± 0.002
**OPC_E_**	23	23	8	15	6	0.87 ± 0.04	0.009 ± 0.001
**OPC_S_**	23	42	17	25	4.36	0.92 ± 0.04	0.0014 ± 0.002
**OPC_P_**	14	16	1	15	4.60	0.59 ± 0.14	0.0070 ± 0.002
**OPC_W_**	11	16	9	7	4.25	0.81 ± 0.1	0.006 ± 0.001
**TOTAL**	89	53	17	36	7.86	0.92 ± 0.01	0.01 ± 0.001

## Supplementary Materials

The following are available online at https://www.mdpi.com/2076-2615/10/9/1594/s1, Table S1: Geographical distribution of Colombian Creole sheep that were sampled for mitochondrial DNA (mtDNA) genotyping, Table S2: Haplotypes from Europe, India, Caucasus and America used in this study for global comparison, Table S3: mtDNA D-loop polymorphic sites in 31 haplotypes of Colombian Creole sheep, Table S4: List of *Ovis aries* haplotypes obtained from statistical parsimony analysis (TCS), with treatment of gaps as a 5th character state and fixed connection limit at 15 steps for the D-loop locus, Table S5: Analysis of molecular variance (AMOVA) and pairwise Fst in five breeds of Colombian Creole sheep, Figure S1: Phylogenetic tree generated from maximum likelihood (IQ-TREE), reconstructed based on the analysis of 31 haplotypes found in Creole sheep from Colombia, Figure S2: Phylogenetic tree generated from maximum likelihood (IQ-TREE), reconstructed based on the analysis of 31 haplotypes of Creole sheep from Colombia and 62 haplotypes of sheep from different continents.
