# Supplementary material for: Mitochondrial DNA Variations in Colombian Creole Sheep Confirm an Iberian Origin and Shed Light on the Dynamics of Introduction Events of African Genotypes
Source: Animals (Basel). 2020 Sep 8;10(9):1594. doi: 10.3390/ani10091594 (PMC7552328; doi:10.3390/ani10091594)
Supplement: Supplementary file 1 [file animals-10-01594-s001.zip › animals-906196-suppl-PROOF/Supplementary Table S4.docx]

Article

Mitochondrial DNA variations in Colombian Creole sheep confirm an Iberian origin and shed light on the dynamics of introduction events of African genotypess

Herman Alberto Revelo, Diana López-Alvarez, Vincenzo Landi, Lauden Rizzo and Luz Angela Alvarez

**Table S4.** List of *Ovis aries* haplotypes obtained from statistical parsimony analysis (TCS), with treatment of gaps as a 5th character state and fixed connection limit at 15 steps for the D-loop locus. Wool Creole sheep (CL), Ethiopian (OPC_E_), Sudan (OPC_S_), Pelibuey (OPC_P_), and Wayúu (OPC_W_).

**Number of haplotypes = 31**

**Number of sequences = 89**

- h1(14): BCL22, BCL20, BCL2, NCL165, OPC_E_39, OPC_S_21, OPC_E_201, OPC_E_200, OPC_E_198, OPC_W_43, OPC_W_26, OPC_W_31, OPC_W_217, OPC_W_111
- h2(2): BCL15, BCL14
- h3(1): BCL13
- h4(2): BCL3, OPC_S_209
- h5(1): BCL1
- h6(1): NCL177
- h7(1): OPC_W_77
- h8(1): OPC_P_271
- h9(1): NCL175
- h10(3): OPC_E_196, OPC_E_207, OPC_W_ 67
- h11(1): NCL172
- h12(2): OPC_S_71, OPC_E_88
- h13(1): OPC_E_92
- h14(2): OPC_S_59, OPC_S_72
- h15(1): OPC_E_73
- h16(1): OPC_E_29
- h17(1): OPC_W_58
- h18(1): BCL12
- h19(2): OPC_P_913, OPC_P_917
- h20(12): OPC_E_186, OPC_S_42, OPC_W_915, OPC_S_231, OPC_P_927, OPC_P_928, OPC_P_920P, OPC_P_918, OPC_P_914, OPC_P_129, OPC_P_136, OPC_E_119
- h21(1): OPC_S_86
- h22(5): BCL18, OPC_E_33, OPC_S_45, OPC_S_208, OPC_W_212
- h23(1): OPC_S_68
- h24(12): BCL10, OPC_S_41, OPC_S_44, OPC_S_57, OPC_E_62,OPC_S_ 70, OPC_S_75, OPC_E_80, OPC_E_C83, OPC_E_194, OPC_W_81, OPC_P_911
- h25 (1): OPC_S_43
- h26 (6): BCL5, CLB4, BCL28, OPC_S_106, OPC_P_134, OPC_P_135
- h27 (1): OPC_S_102
- h28 (8): OPC_E_97, OPC_E_53, OPC_E_61, OPC_S_69, OPC_E_76, OPC_E_C77, OPC_E_78, OPC_S_203
- h29 (1): OPC_P_105
- h30 (1): OPC_E_34
- h31 (1): OPC_S_46

| 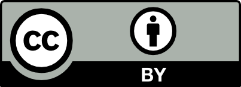 | © 2020 by the authors. Licensee MDPI, Basel, Switzerland. This article is an open access article distributed under the terms and conditions of the Creative Commons Attribution (CC BY) license (http://creativecommons.org/licenses/by/4.0/). |
| --- | --- |
