# Supplementary figures and images for: Mitochondrial DNA Variations in Colombian Creole Sheep Confirm an Iberian Origin and Shed Light on the Dynamics of Introduction Events of African Genotypes
Source: Animals (Basel). 2020 Sep 8;10(9):1594. doi: 10.3390/ani10091594 (PMC7552328; doi:10.3390/ani10091594)

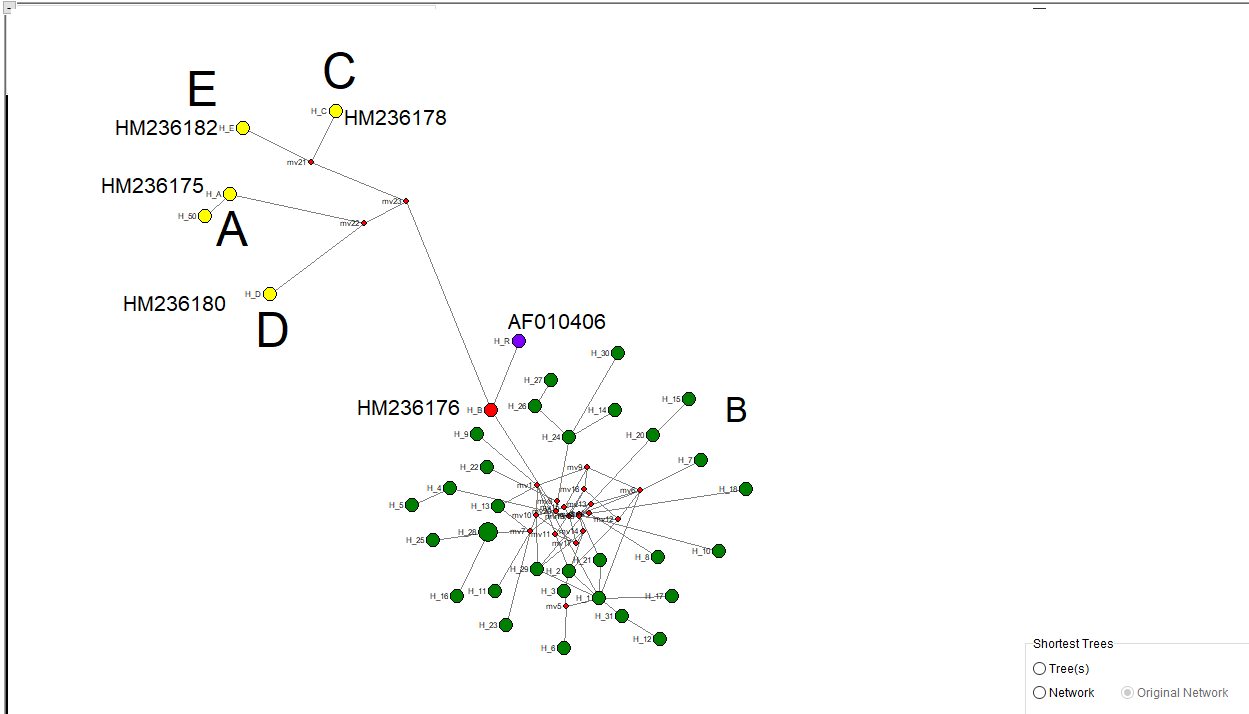

Supplement: Supplementary file 1 [file animals-10-01594-s001.zip › animals-906196-suppl-PROOF/Supplementary Figure S1.bmp]

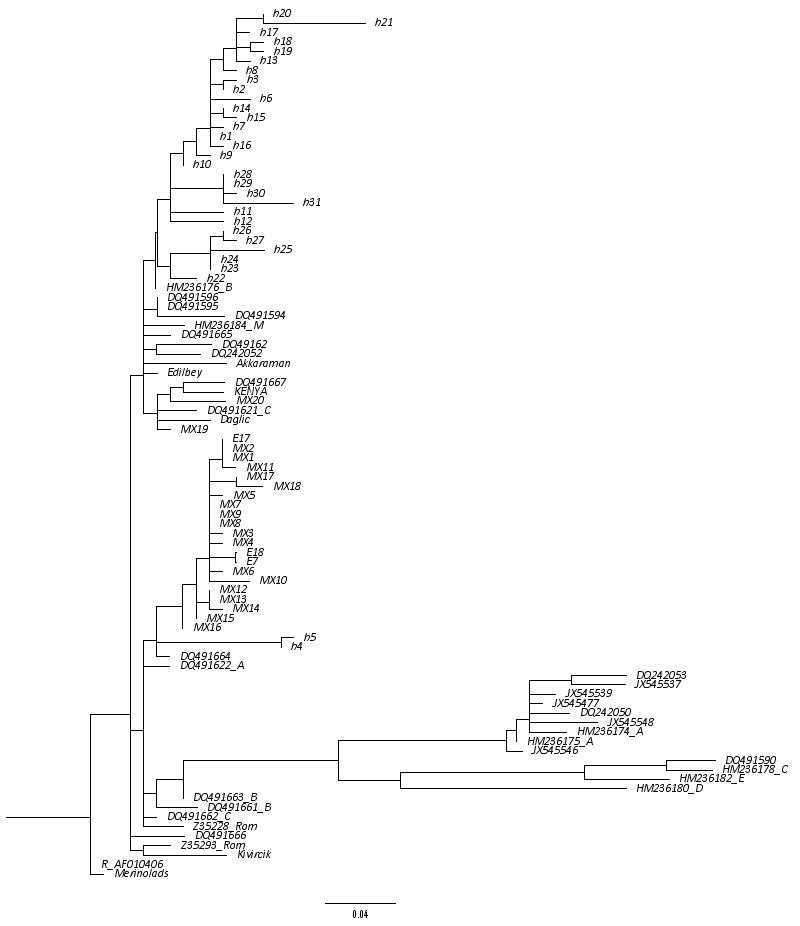

Supplement: Supplementary file 1 [file animals-10-01594-s001.zip › animals-906196-suppl-PROOF/Supplementary Figure S2.jpg]
